# Extracellular Vesicles as Biomarkers of Systemic Lupus Erythematosus

**DOI:** 10.1155/2015/613536

**Published:** 2015-09-07

**Authors:** Javier Perez-Hernandez, Raquel Cortes

**Affiliations:** ^1^Genotyping and Genetic Diagnosis Unit, INCLIVA Biomedical Research Institute, Accesorio 4, Avenida Menendez Pelayo, 46010 Valencia, Spain; ^2^Research Group of Cardiometabolic and Renal Risk, INCLIVA Biomedical Research Institute, Accesorio 4, Avenida Menendez Pelayo, 46010 Valencia, Spain

## Abstract

Systemic lupus erythematosus is an autoimmune disease that predominantly affects women and typically manifests in multiple organs. The damage caused by this disorder is characterized by a chronic inflammatory state. Extracellular vesicles (EVs), including microvesicles (also known as microparticles), apoptotic bodies, and exosomes, are recognized vehicles of intercellular communication, carrying autoantigens, cytokines, and surface receptors. Therefore, the evidence of EVs and their cargo as biomarkers of autoimmune disease is rapidly expanding. This review will focus on biogenesis of extracellular vesicles, their pathophysiological roles, and their potential as biomarkers and therapeutics in inflammatory disease, especially in systemic lupus erythematosus.

## 1. Introduction

Systemic lupus erythematosus (SLE) is an autoimmune disease, characterized by its clinical heterogeneity and effect on several organs since it has a wide profile of autoantibodies [1, 2]. The prevalence of SLE varies from 20 to 150 cases per 100,000 of population, with a high prevalence in women (9 : 1) [3]. Despite being currently incurable, in recent decades, survival rates and longevity have increased due to improvements in therapies and diagnosis. Thus, it has a high impact on long-term medical costs associated with frequent cycles of disease flare and remission [4].

As are many autoimmune disorders, SLE is a multifactorial disease in which genetic and environmental factors interact to modulate the final phenotype. Some loci have been associated with an increase in the risk of SLE (complement components C1q and C4) while others are generally related to several autoimmune diseases, such as diabetes or rheumatoid arthritis (e.g., PTPN22 and STAT4) [5, 6]. Moreover, an epigenetic dysregulation, found in many SLE patients, has been proposed as crucial in the initiation and progression of the disease. Thus, several studies concerning DNA methylation [7, 8], histone acetylation [9, 10], and microRNAs [11, 12] have evidenced epigenetic cross talk [13]. Furthermore, environmental factors (Epstein-bar virus and pesticides) and hormones may trigger autoimmune responses and modulate the alternating periods of SLE flares [14].

One of the most affected organs in SLE is the kidney. The deposition of immune complexes, activation of complements and macrophages, and production of proinflammatory cytokines and chemokines lead to lupus nephritis (LN). Present in almost two-thirds of SLE patients during their lifetime [15, 16], up to 30% of patients progress to end-stage renal failure [17]. In particular, clinical manifestations of active LN include proteinuria, active urinary sediments, and progressive renal dysfunction [18]. Currently, the invasive procedure of renal biopsy provides a direct visualization of renal affection. A recent work, however, shows no correlation between clinical and histological remission, which discards this procedure as a prognostic biomarker [19].

Despite being well established and easy to measure, complements C3 and C4, proteinuria, anti-dsDNA, or creatinine clearance is not as specific or as sensitive as desired. Currently, the SLE Disease Activity Index (SLEDAI) is the most commonly used indicator. It consists of a list of 24 items of which sixteen are clinical variables and eight are laboratory tests such as urinalysis, blood complement levels, increased anti-DNA antibody levels, and low platelet and white blood cell counts. A final score of 6 or higher seems to be consistent with an active disease state [20].

Despite improvements in the diagnosis and prevention of SLE flares, laboratory markers are still unsatisfactory. Over the last few years, the extracellular vesicles (EV), which carry nucleic acids, proteins, and lipids, have been described as essential players in several cellular processes [21, 22]. EVs are small membranous vesicles, ranging from 30 nm to 5 *μ*m, and receive different names depending on their biogenesis and origin. Usually, they are classified as exosomes, microvesicles/microparticles, and apoptotic bodies.

This review focuses on the role of extracellular vesicles (EV) as biomarkers to assess disease activity and the response to therapy in SLE.

## 2. Extracellular Vesicle Biogenesis and Characteristics

Extracellular vesicles, small membranous spherical structures composed of a lipid bilayer, are released by different kind of cells and found such biofluids as urine, plasma, saliva, CSF, synovial fluid, and breast milk [23]. These vesicles can be released by different kinds of cells and carry DNA, coding and noncoding RNAs, proteins, and lipids [21, 22]. Profiling of EV-associated RNA has shown important differences with parental cellular RNA [24]. Moreover, RNA species (miRNA or mRNA) shuttled by EVs maintain their function when transferred to the recipient cells, suggesting epigenetic signaling and an important role in cell-to-cell communication [25].

The general term “EV” includes different types of vesicles. They are not homogeneous and overlapping in size and are classified according to different parameters, biochemical composition, morphology, biogenesis, and size [26, 27] (Table 1). Exosomes are the smallest vesicles (30 nm to 150 nm in diameter), derive from the inward budding of endosomes, and accumulate in intraluminal vesicles known as multivesicular bodies. These EVs are released to the lumen by exocytosis [28]. Microvesicles or microparticles (also referred to as shedding vesicles, ectosomes, or prostasomes) are generally larger than exosomes (100 nm to 1000 nm) and include all structures created by budding and fission directly from the plasma membrane [29]. Finally, apoptotic bodies are released as the consequence of apoptosis, and their diameters vary from 1000 nm to 5000 nm. They are also produced by direct budding of the membrane when cells suffer apoptosis (Table 1).

Currently, there is no consensus on a gold-standard method to isolate/purify EVs according to the type of biofluid or EV type desired [30]. That, notwithstanding, differential ultracentrifugation is the method most frequently mentioned in the literature. It is based on a two-step protocol that begins with a low-speed centrifugation at 10,000–17,000 ×g, which separates apoptotic bodies and larger EVs, and a second step at a higher speed, 100,000–200,000 ×g, depending on the study and the average size of EV required [31]. Alternatively, EVs can be isolated using immunoaffinity beads against surface proteins, filtering the sample through a nanomembrane, utilizing commercial products for exosome enrichment, or employing size-exclusion chromatography principles [32–35].

According to the International Society for Extracellular Vesicles (ISEV), most EV preparations are heterogeneous. Over the last few years, there have been great efforts made to establish appropriate guidance for EV isolation and characterization with minimum experimental requirements [30]. Furthermore, three public databases contain updated information about EVs: EVpedia, ExoCarta, and Vesiclepedia [36–38].

## 3. Extracellular Vesicles in Inflammatory Disease

The damage caused by most autoimmune disorders is characterized by a chronic inflammatory state; so the regulation of inflammation becomes essential in order to ameliorate a patient's condition.

**Figure 1:**
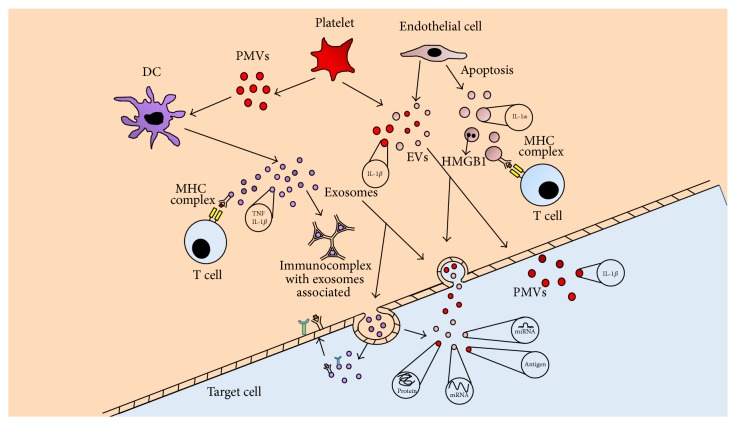
Role of extracellular vesicles in inflammation. Extracellular vesicles (EVs) from mature dendritic cells (DC) provide antigen to T cells and promote a proinflammatory response, mediated by host factors present within exosomes and apoptotic bodies (TNF, HMGB1, etc.). Autoantigens in EVs are recognized by autoantibodies and form immune complexes. Platelet-derived microvesicles (PMVs) activate DC and carry IL-1*β*. EVs in target cell can be involved in antigen presentation and the transfer of major histocompatibility complex (MHC) molecules and antigens, participating in immune regulation. Finally, EVs activate or transfer surface receptors and deliver various RNA species (including mRNA and small RNAs) to target cells. DC: dendritic cell, EVs: extracellular vesicles, MHC: major histocompatibility complex, and PMVs: platelet-derived microvesicles.

Recently, few studies have been conducted in order to establish the relationship between damage-associated molecular patterns (DAMPs) and EV transport. These are endogenous molecules found normally inside cells such as histones, purine metabolites, and mitochondrial components. Under cellular stress or injury conditions, however, DAMPs are released into the extracellular space by damaged tissues, thus activating innate immune cells [39]. Therefore, they are likely to play a determinant role in the appearance and persistence of inflammation. Some DAMPs have been characterized inside EVs as nuclear HMGB1, high mobility group protein B1, ATP (inside apoptotic bodies), or S100 proteins (group of ligands of toll-like receptors) (Figure 1) [40, 41].

Moreover, the EV transport of cytokines and chemokines has emerged as an interesting mechanism for the spread and maintenance of inflammation. For instance, rheumatoid arthritis patients present platelet-derived microparticles with an abundance of IL-1*β*, which induces cytokine release from synovial fibroblasts [42]. Other authors have shown apoptotic bodies carrying active forms of CX3CL1/fractalkine and stimulating chemotaxis in macrophages [23, 43].

Similarly, microvesicles found in the synovial fluid of rheumatoid arthritis patients form proinflammatory immune complexes which may work as autoantigens and autoadjuvants, initiating and perpetuating autoantibody production (Figure 1) [44, 45]. Moreover, in juvenile idiopathic arthritis, synovial exosomes released by macrophages transport a nuclear phosphoprotein named DEK. This protein, involved in chromatin organization, tends to form high affinity complexes with IgG2, which results in joint inflammation [46].

Many studies have pointed out the defective clearance of apoptotic bodies and their subsequent accumulation as a main source of autoantigens in SLE. This results in both chronic organ and tissue damage, as well as the development and maintenance of the systemic autoimmune disease [47, 48]. Recently, circulating microparticles of SLE patients have been associated with particular clinical features, and specific protein patterns have been found. This suggests the importance of EV in driving pathological responses [49–51]. In addition, major ribonucleoproteins antigenically active for lupus and Sjogren's syndrome have been found in salivary gland-derived exosomes [52].

## 4. Extracellular Vesicles as Biomarkers of Systemic Lupus Erythematosus

The study of circulating microparticles (MP) in the plasma of SLE patients has outlined novel subpopulations of platelet, endothelial, and leukocyte-derived MP, some of which have clinical and serological correlations. Cytometry studies performed by Nielsen et al. showed correlations between a population of MP of endothelial origin (AnxV-CDMPs) and disease activity measures, glomerulonephritis, and vascular dysfunction [49]. Thus, the phenotype of endothelial MP offers strong potential as a specific biomarker of vascular pathology associated with SLE. Confirming this hypothesis, Parker et al. have shown an increase of endothelial MP with active SLE when compared to controls. Immunosuppressive therapy reduced the cardiovascular risk by reducing the number of circulating endothelial MP [53].

Moreover, the protein signature of these MP reveals specific patterns that could be used as biomarkers of the activity and progression of SLE. Østergaard et al. have shown a special spectrum of MP in SLE patients, with a particularly unbalanced and decreased microtubule and cytoskeletal composition, which differs from healthy individuals or even rheumatoid arthritis patients [50]. Therefore, the amounts and characteristics of circulating MPs provide new targets for assessing SLE pathogenicity and treatments.

Nevertheless, lupus nephritis (LN) is still a major cause of the morbidity and mortality of SLE with 10–30% of all cases progressing to end-stage renal disease [17]. The investigation of new biomarkers to assess glomerular damage without invasive biopsy has become essential in order to monitor disease progression.

In that sense, urine is the ideal biological fluid for new biomarkers because of its uncomplicated and noninvasive collection. Since the identification and characterization of urinary exosomes by Pisitkun et al., 2004 [54], many studies focusing on urinary exosomes as a source of biomarkers in renal, systemic, and urogenital diseases have been performed [55–58].

Some EV-associated miRNAs, small noncoding RNAs that modulate gene expression, have been proposed as biomarkers of kidney damage in SLE. Over the last few years, the characterization of exosomal miRNA, as opposed to nonexosomal miRNA, by deep sequencing has confirmed the notion of urinary exosomes as a stable source of miRNA biomarkers [59]. Particularly, Ichii et al. have shown an increase in the levels of miR-26a in exosomes from patients of LN and a positive correlation with urinary protein levels, suggesting its convenience as a predictive biomarker of podocyte injury [60]. Similarly, Solé et al. have reported reduced levels of miR-29c in LN patients when compared to controls. Moreover, those levels correlated with renal function and the degree of renal fibrosis, highlighting a potential role in predicting histological fibrosis [61].

Especially relevant is the study of miR-146a, reported to be markedly downregulated in PBMCs, contributing to alterations in type 1 interferon (IFN) pathway in patients with SLE [62]. Furthermore, several studies have assessed its biomarker relevance and found it downregulated as well in plasma and serum [63, 64] but overexpressed in the glomeruli of LN patients [65]. Moreover, some SNPs have been correlated with a lower expression of miRNA in a case-control study in Europeans [66]. Finally, our group has found much higher levels of miR-146a inside urinary exosomes compared to whole urine or exosome-depleted fractions, especially in patients with active LN (data under review).

Regarding messenger RNA cargo, similar studies have been performed to find new markers of kidney damage. Recently, urinary exosome levels of CD2AP mRNA (protein participating in the glomerular filtration barrier) were found downregulated in patients and correlated with proteinuria and severity of renal fibrosis [67]. Similarly, the induction of podocyte damage in rats showed an increase of cystatin C mRNA levels in exosomes, which was representative of glomerular damage, correlating with renal mRNA and protein expression [68].

Finally, protein levels of the adhesion molecule ADAM10 were found higher in the exosomes of patients with glomerular disease, including LN [69]. An important substrate of this protein is the Notch receptor, not only involved in podocyte development but also playing a role in glomerular disease [70]. Moreover, transcription factors related to early podocyte injury were found in urinary EVs, but not in the whole urine of acute and chronic renal patients [57].

Thus, the analysis of urinary exosomes could be considered a reliable, noninvasive approach to the physiological state, offering complementary information to the invasive kidney biopsies. Exosomes are likely to replace biopsies in the future.

## 5. Extracellular Vesicles as a Therapeutic Approach

Leaving aside their promising future as biomarkers, EVs and their cargo could be exploited for therapeutic purposes in a broad range of procedures.

Due to the fundamental role of EVs in regulating biological processes and promoting inflammation and tumor growth under pathophysiological conditions [71], therapeutic actions are being developed to reduce the load of circulating EVs using different strategies: by inhibiting EV formation and release, by blocking EV-specific components with small interfering RNA, and by inhibiting EV uptake [72, 73]. Although reducing the amount of apoptotic bodies or MPs is especially attractive for autoimmune disorders with a high inflammatory component such as SLE, interfering with the general aspects of biogenesis could lead to undesirable off-target effects. Therefore, such actions would require a targeting system capable of selecting EV-cell specific populations.

Nevertheless, there has been a rapid increase in the number of studies investigating the role of EVs in the modulation of the immune system. Thus, EVs containing anti-inflammatory substances could be used as therapeutic agents to promote immunosuppressive responses. Some studies have shown these types of compounds to have a longer half-life when encapsulated in EVs, increasing the survival of mice after LPS-induced septic shock [74]. Therefore, these vesicles could work as immunomodulatory agents for autoimmune, inflammatory, and hypersensitivity disorders [23, 75].

Several studies have been performed in order to examine the immunomodulatory action of dendritic cell (DC) derived exosomes. For instance, bone marrow-derived DCs were treated with IL-10, and the reduction of autoimmunity was evaluated in some murine models of disease, such as collagen-induced arthritis and delayed hypersensitivity [76, 77]. Further work, however, will be required before clinical translation is possible in order to optimize cell types for the production of well-defined EV therapeutic agents that are safe in the long run.

Regarding drug delivery, patient-derived EVs could be used to package molecules capable of avoiding immune responses. Exosomes are ideal for transfer purposes and may become a strong delivery tool for pharmacological agents. Because of their bilipidic structure, they are flexible vectors with the ability to carry select nucleic acids (miRNA, siRNA, and mRNA), proteins, and active chemical drugs across biological barriers [74, 78, 79].

Altogether, this information underscores the broad potential of EV for the treatment and prevention of flares in autoimmune disorders like SLE. Nonetheless, further investigation is needed to elucidate the precise effect of EV treatment with immunomodulatory purposes.

## 6. Concluding Remarks

Extracellular vesicles have emerged as important “nanoshuttles” of information between cells, carrying proteins, genetic information, and bioactive lipids to modify the phenotype and function of recipient cells. EVs are potential regulators in autoimmune disorders, having a determinant role in the appearance and maintenance of inflammation. In SLE, the defective clearance of apoptotic bodies and their accumulation represents a major source of autoantigens. The presence of EV-specific patterns and their cargo as biomarkers of SLE activity and progression is rapidly expanding. In that sense, miRNAs, mRNA, and proteins transported into urinary exosomes are representative of glomerular damage, correlating with proteinuria and the severity of renal fibrosis in lupus nephritis. Still, the precise pathophysiological functions of these vesicles and their role as therapeutic agents or targets are not fully understood. Although further studies are necessary, we foresee a great potential for EVs as immunomodulatory agents and therapeutic vehicles in the future.

## Figures and Tables

**Table 1:** Key features of extracellular vesicles.

EV types	Size	Biogenesis	Markers	Contents
Exosomes	30–100 nm	Endolysosomal pathway. Released by exocytosis of multivesicular bodies	Tetraspanins (CD63, CD9, and CD81), Alix, and TSG101	miRNAs and mRNA; lipids, DNA membrane proteins and lipids, cytokine receptors, and MHC molecules

Microvesicles/microparticles	100–1000 nm	Cell surface. Outward budding of plasma membrane	Integrins, selectins, and CD40 ligand	mRNA, noncoding RNAs, membrane proteins, receptors, and cytoplasmic proteins

Apoptotic bodies	Up to 5000 nm	Cell surface. Released from cellular blebs during apoptosis	Phosphatidyl-serine	Nuclear fractions, cell organelles, DNA, rRNA, and mRNA

EV: extracellular vesicles, MHC: major histocompatibility complex, mRNA: messenger RNA, miRNA: microRNA, rRNA: ribosomal RNA, and TSG101: tumor susceptibility gene 101.
